# A short history of innate immunity

**DOI:** 10.1590/0074-02760230023

**Published:** 2023-05-08

**Authors:** Yuri Chaves Martins, Flávia Lima Ribeiro-Gomes, Cláudio Tadeu Daniel-Ribeiro

**Affiliations:** 1 Saint Louis University School of Medicine, Department of Anesthesiology, Saint Louis, MO, USA; 2 Fundação Oswaldo Cruz-Fiocruz, Instituto Oswaldo Cruz, Centro de Pesquisa, Diagnóstico e Treinamento em Malária, Laboratório de Pesquisa em Malária, Rio de Janeiro, RJ, Brasil

**Keywords:** immune response, inflammation, innate immunity, history, modern immunology

## Abstract

Innate immunity refers to the mechanisms responsible for the first line of defense against pathogens, cancer cells and toxins. The innate immune system is also responsible for the initial activation of the body’s specific immune response (adaptive immunity). Innate immunity was studied and further developed in parallel with adaptive immunity beginning in the first half of the 19th century and has been gaining increasing importance to our understanding of health and disease. In the present overview, we describe the main findings and ideas that contributed to the development of innate immunity as a continually expanding branch of modern immunology. We start with the toxicological studies by Von Haller and Magendie, in the late 18th and early 19th centuries, and continue with the discoveries in invertebrate immunity that supported the discovery and characterization of lipopolysaccharide (LPS) and pattern recognition receptors that led to the development of the pattern recognition and danger theory.

Innate immunity is one of the two major branches of modern immunology. Innate immune mechanisms are essential to secure cellular integrity, homeostasis, and survival of all living organisms.^1^ ^,^ ^2^ Notwithstanding, as a consequence of being involved in tissue homeostasis, the innate immune system is responsible for the first line of defense against pathogens, cancer cells and toxins prior to the activation of the body’s specific immune response (adaptive). Classically, innate immune recognition and response are described as fast, non-specific, non-adaptive and memory-free.^3^ Of these four characteristics, being fast is the only innate immune feature that was not challenged until now. However, regardless of its traits, innate immune mechanisms are exceptionally effective insofar as severe infections, neoplasia and poisoning are quite rare.

Despite the innate immune system being changed only on an evolutionary time scale by the selective pressures that microbes impose,^4^ it has been refined for a much longer period of time than the adaptive immune system. Protozoans, all multicellular animals (metazoans) and plants have an innate immune system, and it represents the only defense system against pathogens in most species.^2^ ^,^ ^5^ Therefore, innate immune systems appeared around 1 billion years ago in the evolutionary process, much earlier than the materialization of adaptive immunity around 450 million years ago in organisms resembling primitive jawed fishes.^2^ In fact, since classically innate immunity had no specificity and memory, it was considered totally independent from adaptive immunity for a long time.^6^

Nevertheless, innate immune systems are of particular importance in vertebrates. The adaptive immune system remembers specific pathogens previously encountered and promptly responds when they are faced again.^7^ However, adaptive immune responses with specific antibody and lymphocyte production require at least a week to develop on first exposure to new pathogens.^3^ By contrast, some pathogens (*Staphylococcus aureus*, *Vibrio cholerae*) have estimated generation times of around one to two hours in the wild, being able to have incubation periods of a single day.^8^ Hence, vertebrates rely on their innate immune system to survive the first critical hours and days of exposure to a new pathogen. In addition, innate immune system activation is required to activate and direct the adaptive immune system most of the time in vertebrates.^9^ As a result, it is now hypothesized that the specificity, antibody maturation, immunological memory, and secondary responses of adaptive immunity allowed higher vertebrates to reduce the number of variants of innate defense mechanisms originating from invertebrates.^2^ ^,^ ^10^ Therefore, vertebrates combine the two arms in an intricate inter-dependent network.

It is consensual that the innate immune system must be capable of doing three things: (1) recognition of a diverse array of aggressions; (2) stopping or mitigating the aggression once it is recognized; (3) sparing self-structures of the host (*i.e.*, there must be self-tolerance) and foreign non-aggressors (*i.e.*, there must be tolerance to commensal and mutualistic organisms).^4^ ^,^ ^9^ Accordingly, innate immunity can be subdivided into two main fields: (a) the sensing (afferent) arm deals with how the system detects aggression including pathogens; (b) the effector (efferent) arm includes the mechanisms that eliminate or tolerate the aggression. Each field can be further divided into physical or anatomic barriers, cellular and humoral components. Studies on both fields of the innate immune system have evolved in parallel with studies on the adaptive immune system and have increasingly garnered importance and emphasis in the field of immunology over the past century.

Like most fields of science, the history of innate immunity oscillates between periods of high change/productivity and relative inactivity. The high active periods are frequently stimulated by a disturbing new observation or hypothesis that opens new landscapes whilst the quiet periods occur when the predictions of old theories have been exhaustively tested.^11^ ^,^ ^12^ ^,^ ^13^ In the present overview we will discuss the historical roots of the main concepts and findings that contributed to the development of innate immunity as a continually expanding branch of modern immunology (Figure). We will not focus on the details of every cell or molecule involved in innate immunity, but on the big picture and big ideas that advanced the field.

**Figure:**
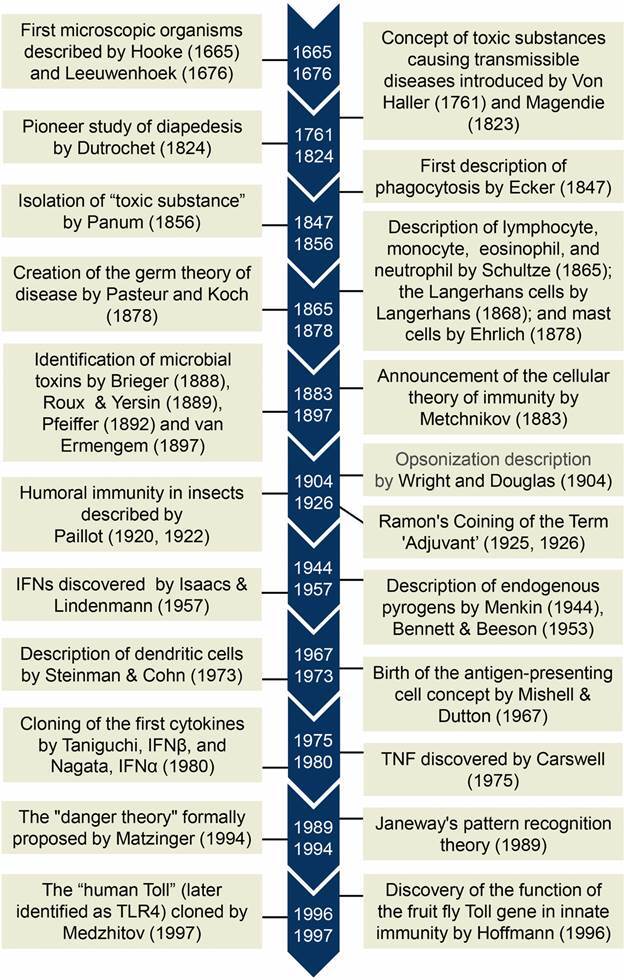
Timeline of important discoveries related to innate immunity. Sequences of discoveries and concepts that have provided the basis for our current understanding of innate immunity. IFN: interferon; TNF: tumor necrosis factor; TLR: toll like receptor.

Early years

“*A good historian can find precedent for everything. But an even better historian knows when these precedents are but curiosities that cloud the big picture*”.^14^

Innate immunity has a strong focus on explaining how organisms deal with infectious diseases. Infectious diseases have been a constant threat to humanity since the first human beings wandered the earth.^14^ Therefore, trying to pinpoint the ideas and discoveries that started this field of immunology is not an easy task and can be a subject of endless debate.

The term innate immunity was defined by Charles Janeway Jr (1943-2003).^3^ Before Janeway’s seminal paper, other terms including ancestral immunity, immediate immunity, natural immunity, and non-specific immunity were used to describe this branch of immunology.^6^ However, it is largely accepted that the creation of what would be called innate immunity developed based on earlier discoveries in toxicology, hematology, and microbial pathogenesis.^4^ ^,^ ^11^

The description of microscopic organisms was first done by Robert Hooke (1635-1703), who described microscopic fungi (1665), and Antonie van Leeuwenhoek (1632-1723), who first described bacteria (1676).^15^ However, both authors did not clearly associate their discoveries with disease pathogenesis. At their time, based on Hippocrates of Cos (c. 460 BC - c. 370 BC), Aulus Celsus (c. 25 BC - c. 50 AD), and Galen of Pergamon (129-216), illness was described as maladjustment of the normal ratios of the four vital humors: the blood (*sanguis*), the phlegm (*pituita*), the yellow bile (*chole*), and the black bile (*melaine chafe*).^11^

The humoral disease paradigm started to change in the second half of the 18th century when Albrecht Von Haller (1708-1777) investigated the toxic properties of decaying tissue (as in the case of gangrene) to determine why it causes disease when ingested or present in living animals (1761).^16^ Von Haller observed that oral ingestion or intravenous injection of putrid fluids obtained from decomposing organic matter, such as putrid fish or meat, were associated with development of fever and other manifestations of illness by experimental animals. As extracts from fresh fish or meat did not produce febrile reactions, Von Haller concluded that a disease-producing toxic principle was formed during putrefaction. Later François Magendie (1783-1855) showed that intravenous injection of decomposed meat to experimental animals caused symptoms of illness (1823).^17^ Therefore, Von Haller and Magendie pioneered the idea that toxic substances can cause transmissible diseases. Nevertheless, these earliest workers did not attempt to purify toxic substances.

Inspired by these ideas, in 1856, Peter Ludvig Panum (1820-1885) succeeded in isolating a substance from infected tissues that he called putrid poison, which could cause septicemia when injected into animals.^18^ These findings were further expanded upon by Ernst von Bergmann (1836-1906),^19^ ^,^ ^20^ who isolated a molecule which he termed sepsin, derived from waste products of beer fermentation that could cause intestinal hemorrhaging and fever when injected into dogs and frogs (1868). It is important to notice that Panum and von Bergmann did not accept at first that putrid poison and sepsin could be derived from bacteria.^20^ ^,^ ^21^ These findings showed that poisonous substances derived from decaying tissue or sick individuals could cause disease and suggested that white blood cells could be involved in the process.

However, this early data could not explain how a limited amount of a toxic substance could be serially transmitted from one individual to the next without losing potency. More than that, the toxic substance appeared to increase in potency in the same individual with the progression of the disease. Jacob Henle (1809-1885) tried to explain this phenomenon with the idea that the toxic substances could multiplicate itself, thereby having attributes of a living organism.^22^ Therefore, Henle stood at a point of transition between the pre-microbial and the microbial eras.^23^

Scientists started to make a connection between microorganisms, especially the ones involved in fermentation, and infectious diseases in the middle of the 19th century. Although Theodor Schwann (1810-1882) revealed that putrefaction was a microbiological process caused by living microscopic organisms in 1837,^24^ Louis Pasteur (1822-1895) was the one to demonstrate, beyond any doubt, that germs, and only germs, were responsible for the decomposition of organic matter.^25^ Pasteur also discovered that two silkworm diseases, pebrine and flacherie, were infectious, contagious, and hereditary.^26^ Robert Koch (1843-1910), a disciple of Henle, famously proved that each infectious disease is caused by a specific living microorganism that was able to enter the body, multiply and produce increasing levels of toxic substances causing its symptoms.^27^ ^,^ ^28^ Concurrently with Koch, Pasteur also showed that microorganisms were necessary and sufficient to cause infectious diseases.^29^

The research spearheaded by Koch and Pasteur is responsible for the creation of the germ theory of disease, which prompted toxicologists to investigate which poisons present in microbes could cause disease.^30^ This was demonstrated for the first time in 1888 by Ludwig Brieger (1849-1919), who coined the term “toxin” and identified putrescine and cadaverin, organic compounds released by bacteria.^31^ Posteriorly, Richard Pfeiffer (1858-1945) succeeded in isolating a substance with the same characteristics as Panum’s putrid poison from pure cultures of *V. cholerae*, which he called endotoxin (from the Greek ‘endo’ meaning ‘within’) (1892).^32^ Pfeiffer also demonstrated that endotoxin could elicit its toxic effects when injected into animals that had previously been immunized against *V. cholera*, concluding, as Brieger, that the toxin was responsible for the symptoms displayed by infected individuals. As expected, living vibrio bacteria could not be isolated from animals injected with endotoxin or heat killed *V. cholerae*. Pfeiffer experiments led him to the conclusion that *V. cholerae* had an alcohol insoluble, heat stable toxin associated with the insoluble part of the bacterial cell.^32^ Concomitantly with Pfeiffer, numerous scientists convincingly showed that pathogens could cause deleterious effects though the secretion of toxins.^33^ Notable examples are the studies of Emile Roux (1853-1933) and Alexandre Yersin (1863-1943) with diphtheria toxin^34^ and Emile van Ermengem (1851-1932) with botulinum toxin.^35^ The identification of microbial toxins was a tremendous milestone in the quest to understand how microbes create disease.

In parallel with these discoveries, scientists tried to understand how hosts defend themselves against the damage caused by pathogens and the toxins produced by them. René Joachim Henri Dutrochet (1776-1847) is credited with the first description of blood corpuscles coming out of blood vessels into the extravascular space during acute inflammation (1824).^36^ Based on his osmosis studies, Dutrochet hypothesized that blood vessels had orifices in their wall that allowed the passage of blood cells. Rudolf Wagner (1805-1864) was the first to describe leukocyte rolling in blood vessels of the webbed feet of a grass frog (1839).^37^ These discoveries were allowed by improvements in microscopes in the early 19th century that permitted microscopy magnification of 400x. Some years later, Gabriel Andral (1797-1876) and William Addison (1802-1881), reporting simultaneously, gave a clear description of leukocyte transmigration and diapedesis in inflamed tissue induced by burning or trauma (1843).^38^ ^,^ ^39^ Addison also speculated that pus cells were blood leukocytes that had passed through the wall of vessels and migrated to injured tissue. These findings were confirmed by Augustus Waller (1816-1870) in 1846.^40^ Waller also showed leukocyte diapedesis after the death of experimental frogs, thus showing that their movement was not driven by blood pressure. Nineteen years later, Max Schultze (1825-1874) first described the four different types of blood leukocyte corresponding to what are now recognized as the lymphocyte, the monocyte, the eosinophil, and the neutrophil.^41^ Posteriorly, Julius Cohnheim (1839-1884), after independently describing diapedeses when working with transparent tissues *in vivo*, was the first to postulate that alterations in the vessel walls of inflamed tissue was responsible for the leukocyte rolling and migration in 1867.^42^ One year later, Paul Langerhans (1847-1888), using a technique taught to him by Cohnheim to stain a sample of human skin with gold chloride, described cells with a dendritic morphology in the epidermis.^43^ These cells, known as “Langerhans cells”, are now known to be dendritic cells, but were originally described as nerve cells due to their morphology. Some years after Cohnheim’s and Langerhans’ work, Rudolf Virchow (1821-1902) added more precision to the phenomenon showing that leukocytes could become transiently adherent and sometimes re-enter the blood flow.^44^ Although Mast cells may have been first observed by Friedrich von Recklinghausen (1833-1910), when he described, in 1863, the presence of granulated cells in unstained connective tissues from various species,^45^ it was Ehrlich who coined the name ‘Mastzellen’ (well-fed cells), to describe these cells in 1878.^46^

The description of the most common types of leukocytes and the phenomenon of diapedesis showed that white blood cells were involved in inflammation, but there wasn’t a consensus on the role of these cells in the disease process. This started to change with the announcement of the cellular theory of immunity by Ilya Ilyich Metchnikoff (1845-1916) in 1883.^47^ Metchnikoff was not the first to describe phagocytosis.^48^ There are more than 30 accounts of cells engulfing particles or other cells before Metchnikoff’s earliest paper about it appeared in 1880.^48^ ^,^ ^49^ The earliest of these reports was made in 1847 by Alexander Ecker (1816-1887), who described the presence of erythrocytes inside rabbit spleen cells.^50^ This first study was followed by two others independently made descriptions by Joseph G. Richardson (1836-1886) and Kranid Slavjansky in 1869. Richardson described salivary white blood cells loaded with “white corpuscles” derived from bacteria.^51^ Slavjansky described the presence of carbon particles within alveolar macrophages.^52^ Giulio Bizzozero (1846-1901), working with an experimental model of aseptic inflammation in the anterior chamber of the eye of rabbits showed that white blood cells were able to engulf other dead white blood cells and erythrocytes and speculated about the phagocytosis of germs in 1872.^53^ Slavjansky findings were confirmed by Wiliam Osler (1849-1919) in 1875^54^ who interpreted them as a reaction from the body to “render harmless what might otherwise be very irritating substances”. Thus, proposing a protective role to phagocytosis. Four years later Koch identified Anthrax bacilli in white blood cells, but he interpreted his finding to mean invasion of host cells by bacterial pathogens. In this view, the white blood cell, far from destroying microbes, would be the vector for their spread facilitating the spread of germs throughout the host.^11^

Consequently, before Metchnikoff published his cellular theory of immunity two opposing schools of thought about the function of inflammation, diapedesis and phagocytosis existed: (a) the inflammatory reaction was detrimental to the organism and contributed to the disease process by causing its symptoms and helping the spread of germs throughout the host or (b) the inflammatory reaction was a beneficial host defense mechanism with phagocytes working as scavengers removing dead and alive microorganisms, foreign particles, and disintegrating cells.

The idea that inflammation was a deleterious reaction with no benefit to the host was the most prevalent at the time, being defended by most pathologists such as Virchow, Koch and Cohnheim.^11^ The value of Metchnikoff was, like Pasteur, to prove beyond doubt and try to convince the world that this view was wrong and that inflammation was a defense mechanism beneficial to organisms.^6^ He proposed that the entire animal kingdom can defend themselves against foreign bodies and pathogens using a universal nonspecific mechanism he called phagocytosis. He also gave the name phagocytes to the specialized ameboid cells of mesodermal origin responsible to engulf and digest cellular debris during embryogenesis that would assume a defensive role in the adult individual.^47^ ^,^ ^55^ Metchnikov also linked inflammation, the formation of pus, and diapedesis of white blood cells with the necessity of phagocytes to get to the place where the injury was occurring to defend the organism. Furthermore, in his theory, Metchnikoff proposed that the phagocyte was the primary cell responsible for inflammation and had a central role in “natural” immunity through phagocytosis and the secretion of antibacterial substances.^56^ Therefore, Metchnikoff was the first to unify in a single theory the direct functions of the innate immune system: (1) rapid detection of microbes, (2) phagocytosis, and (3) antimicrobial activity.^57^

Metchnikoff’s cellular theory of immunity suffered fierce opposition, mostly from German pathologists, after its proposal in the 1880s. This resistance was mainly based on serotherapy studies showing that humoral factors were involved in host resistance and immunity to infection. In fact, Emil von Behring (1854-1917) and Shibasaburo Kitasato (1853-1931) demonstrated that immune sera from some vertebrates were protective against diphtheria and tetanus in 1890.^58^ In 1894, Pfeiffer showed the destruction of cholera vibrios inoculated into the peritoneum of a vaccinated guinea pig by a humoral factor without the intervention of macrophages, which was called the Pfeiffer phenomenon.^59^ Pfeiffer was the first to use the term Antikörper (antibody) to characterize this factor and Behring’s antitoxins.^6^ The discovery of non-specific anti-bacterial factors and specific antibodies against different microorganisms and nonbacterial toxins starting in 1888 also supported that humoral factors were involved in host protection against infections. The question of whether immunity to infection was due to cellular or humoral mechanisms still raged on thought the early 20th century.^11^ The cellular theory of immunity had the advantage of better explaining the duration of immunity, as it was difficult to imagine that a humoral factor could persist for a long period, ensuring the protection of the individual. However, because the humoral conception of immunity had the advantage of responding much more logically to the specificity of the immune response often observed, Ehrlich formulation of the side-chain theory of antibody production gave the victory to the humoralists.^60^

Early 20th century

Although both Metchnikoff and Ehrlich received the Nobel Prize for their observations in 1908, the study of innate immunity in vertebrates was eclipsed by findings that have driven adaptive immunity during the first half of the 20th century. During this period, most vertebrate immunologists considered innate immunity to be an unsophisticated part of the immune system whose main purpose was to initiate an adaptive immune response.^11^ However, innate immunity was ignored, but not forgotten and a couple of key discoveries were made during this time that are worth noticing.

Edward Wright (1861-1947) and Stewart Douglas (1871-1936) described opsonization in 1904.^61^ Wright records in his paper “The body fluids modify bacteria in a manner which renders them a ready prey to phagocytes”. Opsonization indicates that substances produced by the host can modulate innate immune responses. Some years later, Gaston Ramon (1886-1963) coined the term adjuvant, to explain why horses immunized with diphtheria toxoid build a stronger antibody response when an abscess develops at the inoculation site or when substances like breadcrumbs or tapioca were mixed with the antigen in the 1920s.^62^ ^,^ ^63^ On the same period, it was observed that diphtheria toxoid vaccine was improved by the addition of alum.^64^ Adjuvants indicate the need to induce an inflammatory reaction at the antigen-injection site to trigger or enhance immune responses.

Studies in invertebrate species, that lack an adaptive immune response, also continued in the period. Although most invertebrate immunologists at the time were influenced by vertebrate immunologists and tried to adequate their discoveries to fit the mainstream humoral or cellular theory of immunity, one exception existed. André Paillot (1885-1944) demonstrated the existence of humoral immunity in insects, unrelated to the type of immunity that was initially thought to be present in all vertebrates in the 1920s.^6^ Paillot performed a series of insightful experiments demonstrating that chemical changes in the insects’ hemolymph could alter and destroy bacteria with minimal or no involvement of phagocytosis. He concluded that invertebrates were able to produce antibacterial substances more rapidly than the antibody production in vertebrates.^65^ ^,^ ^66^ Therefore, Paillot showed that innate immunity could also be mediated by humoral factors, unrelated to vertebrate antibodies, synthesized by the insect following an injection of insect pathogenic bacteria. Vladimir Zernoff (1904-?) showed that it is possible to transfer immunity from one caterpillar to another (passive immunity) in 1927.^67^ The transfer of immunity occurred with use of leukocytes or hemolymph, also showing that passive immunity could be obtained in invertebrates without the use of cells.

Post-war era

The development of new technologies after World War II led to progress in studies of innate immunity. The work of north American scientists including June M Stephens, June Stephens Chadwick, John D Briggs (1926-2002) and Richard E Gingrich in the late 1950s confirmed Paillot’s results and it then became clear that antibacterial factors of low specificity unrelated to antibodies were quickly synthesized by invertebrates following a bacterial infection conferring protection for several days and allowing passive transfer of immunity.^68^ ^,^ ^69^ ^,^ ^70^ ^,^ ^71^

The initial characterization of cytokines also started during this period. The proposal to use the term “cytokine” to name factors secreted by different types of cells involved in inflammatory reactions, was made by Cohen et al. in 1974.^72^ However, the first observations on the existence of endogenous soluble factors capable of modulating reactions in mammalian hosts were made by Valy Menkin (1901-1960) in 1944^73^ and confirmed by Ivan L Bennett Jr (1922-1990) and Paul B Beeson (1908-2006) in 1953.^74^ They observed the presence of an endogenous pyrogen, described as a “fever-promoting substance”, in acute inflammatory exudates. Bennett and Beeson also found that endogenous pyrogens were heat-sensitive and produced by polymorphonuclear leukocytes.

The discovery of interferons (IFNs) followed soon after, in 1957. While investigating the phenomenon of “viral interference”, Alick Isaacs (1921-1967) and Jean Lindenmann (1924-2015) observed the release of an interfering factor from chick chorioallontoic membranes infected with heat- or UV- inactivated influenza virus; this factor was able to interfere with the replication of live virus and they named it interferon.^75^

The history of cytokines continues with the discovery that these fever-promoting and viral interference molecules are also produced by lymphocytes besides other cells. This was only possible after the identification of lymphocytes as immunocompetent cells by James Gowans (1924-2020),^76^ the development of techniques for polyclonal activation of lymphocytes in culture by Peter Nowell (1928-2016)^77^ and of mixed allogeneic lymphocyte culture (MLR) by Bain et al.^78^

Thus, between the years 1964 and 1969, four discoveries, made in the supernatant of *in vitro* antigen and alloantigen-stimulated lymphocyte cultures, set cytokine studies in motion: (1) detection of mitogenic factors by Shinpei Kasakura and Louis Lowenstein;^79^ ^,^ ^80^ (2) detection of a macrophage migration inhibitory factor (MIF) as a molecule derived from activated T cells during delayed-type hypersensitivity;^81^ ^,^ ^82^ and (3) detection of a cytotoxic factor, called lymphotoxin;^83^ ^,^ ^84^ (4) the first report of a substance with chemotactic function were made by Ward et al. showing that stimulated lymphocytes produced chemoattractant factors for monocytes.^85^

Initially, there was great interest from immunologists in the mitogenic factors discovered by Kasakura and Lowenstein in 1965.^79^ Julius Gordon and Lloyd MacLean (1925-2015) demonstrated that the production of these mitogenic factors could be inhibited by puromycin or 5-fluorouracil, suggesting they were derived from lymphocytes.^86^ Posteriorly, in 1969, Dumonde et al. proved antigen-activated lymphocytes as the source of these mitogenic factors and coined the term “lymphokines” to name them.^87^

In the early 1960s, George Mackaness (1922-2007) also introduce the concepts of macrophage activation by showing that inactivation of *S. aureus* and *Listeria monocytogenes* was better achieved in convalescent mice thanks to the presence of resistant macrophages generated during the primary infection.^88^ ^,^ ^89^ He also used electron microscopy to show that macrophages from mice immunized with *L. monocytogenes* had structural differences when compared to macrophages from naive mice.^90^ Mackaness also described cellular cooperation between immune cells by showing that macrophage activation results from specific interactions with sensitized lymphoid cells and the microorganism.^91^ In parallel, James Hirsch (1922-1987) further clarified the mechanism of phagocytosis by describing the fusion between the granule membrane of polymorphonuclear leukocytes and the invaginated cell membrane overlying the ingested particle with discharge of granule content into the phagocytic vacuole.^92^ ^,^ ^93^

There was progress in the chemical characterization of endotoxin due to the development of suitable extraction procedures during the 1950s and 1960s as well. Otto Lüderitz (1920-2015) and Otto Westphal (1913-2004) designated their largely protein-free purified endotoxin product as lipopolysaccharide (LPS), because of the presence of polysaccharide and lipid components.^23^ Largely through the work of Mary Jane Osborn (1927-2019) and Hiroshi Nikaido (1932 - ), LPS was chemically characterized and shown to be part of the cell wall of virtually all Gram-negative bacteria.^23^ Subsequently, it was shown that LPS had more than just toxic properties. In small doses, LPS also exerted an adjuvant effect, helping to activate immune responses against antigens injected in conjunction with LPS.^94^ Furthermore, the presence of LPS is also required to combat and contain the initial stages of Gram-negative bacterial infections.^95^ ^,^ ^96^ ^,^ ^97^

From pharmacology studies, it has been known, since the turn of the 20th century, that substances that produce a biological effect at dilute concentrations, such as LPS, often function by interacting with specific, high-affinity receptors, which are linked generally to a signal-amplification system. Therefore, the questions that followed the first studies with endotoxin concerned the receptor responsible for recognizing it and generating its biological effects. This situation improved somewhat in 1954 when it was shown that lipid A, the lipid portion of the LPS molecule, was responsible for the inflammatory and toxic effects of LPS.^98^ Posteriorly, random chance advanced the characterization of LPS. Between 1960 and 1965, a spontaneous mutation was detected in a mouse line (C3H/HeJ) from the famous Jackson Laboratory in the USA, which resulted in this line becoming resistant to the effects of LPS (for review see^99^). However, a 100% homogeneous solution of LPS was not available at the time, making it difficult to further characterize its receptor.

Finally, at the beginning of 1960s, Nossal et al. described that injection of antigens labelled with iodine-131 into the hindfoot pads of rats migrated to spaces between lymphocytes inside lymph nodes and spleen.^100^ Because the labeling was found between and not inside lymphocytes, the authors speculated that the antigen was present in cytoplasmic extensions of specialized macrophages that would be involved in the production of antibodies. Posteriorly, Robert Mishell (1934-2008) and Richard Dutton described that mononuclear phagocytes from mouse spleen could induce antibody production from naïve lymphocytes *in vitro*.^101^ The authors then confirmed that these cells could prime the adaptive immune system by activating lymphocytes and stimulating antibody production.

From 1970s until now

Starting in the 1970s, new discoveries in the realm of cytokines, endotoxin characterization and antigen presenting cells fueled conceptual innovations in innate immunity. As early as 1971, Nathan et al. identified that the supernatant of stimulated lymphocyte cultures containing MIF had macrophage-activating factor (MAF) activity, which stimulates several functions in macrophages, such as tumoricidal and microbicidal activity.^100^ In the following years, several studies appeared that erroneously credited MIF activity to IFNγ.^101^ Nevertheless, MAF activity has been attributed to IFNγ.^102^ ^,^ ^103^ Almost in parallel (1972), Igal Gery, Richard Gershon and Byron Waksman described that activated macrophages produced a lymphocyte-activating factor that had pyrogenic activity.^102^ ^,^ ^103^

The discovery of the Tumor Necrosis Factor (TNF) followed in 1975, when Carswell et al.^104^ identified in rabbits infected with Bacillus Calmette-Guérin and subsequently challenged with LPS, a serum factor with cytotoxic activity *in vitro* and leading to tumor necrosis. Unlike the factors previously described as lymphocyte-derived products, TNF was a factor derived predominantly from cultured endotoxin-stimulated macrophages. Because TNF, similar to LPS itself, caused fever, diarrhea, shock, and death when injected into mice, it was hypothesized that TNF and lymphocyte-activating factor might be endogenous mediators of endotoxicity.^23^ We now know that cytokines are not only involved in leukocyte activation and fever in sepsis and parasitic diseases, but also in many processes, such as cell differentiation, development, maintenance of homeostasis, etc.

Posteriorly, the development of high-performance liquid chromatography, microsequencing, and molecular biology techniques allowed the purification and sequencing of cytokines secreted in culture supernatants, as well as the cloning of cytokines, the expression of their recombinant forms, and the identification of new cytokines. Currently several chemoattractant cytokines have been cloned and are known as chemokines. With the generation of knockout mice technology in the late 1980s, it was possible to observe that TNF is an important cytokine in the inflammatory process and that, among other things, the anti-tumor effects of TNF are based on its ability to stimulate endothelial cells to produce clotting factors and consequently resulting in blood vessel occlusion and necrosis.^105^

The number of interleukins reached 30 at the beginning of the 21st century. IL-41, a novel immunoregulatory cytokine highly expressed in psoriatic skin, is currently the most recently named cytokine.^106^ But several other cytokines, such as TNF, IFN and some colony stimulating factors (CSFs) escape renaming. Nowadays we know that when we talk about a specific cytokine we may be referring not only to a single protein (derived from the expression of a single gene), but to a family of cytokines, such as the TNF family, the IL-1 family, the chemokine family, etc. Each family has a diverse number of members (the product of a separate gene) and multiple biological properties.^107^

The 1970s and 1980s are also distinguished by the discovery and characterization of dendritic cells. In 1973, Ralph Steinman (1943-2011) and Zanvil Cohn (1926-1993), during microscopic study of the mononuclear phagocytes from mouse splenocytes, described a small population of cells with unique stellate morphology, and named them dendritic cells.^108^ Some years later, Steinman together with Margaret Witmer showed that dendritic cells were orders of magnitude more potent than macrophages and other antigen presenting cells (APCs) in stimulating lymphocytes.^109^

Together with the discovery of cytokines and dendritic cells, that showed that immune and non-immune cells could communicate and influence the behavior of each other, the quest for the LPS receptor continued. Then, in 1978, reports showed that the gene responsible for resistance to LPS, called Lps^d^, was located on chromosome four of C3H/HeJ animals, suggesting that a protein produced by the host, possibly a receptor which could bind to the molecule, was responsible for its biological effects (for review see^110^). At the same time, it was shown that other substances derived from microorganisms, such as unmethylated DNA (present in microbes and different from our DNA, which is predominately methylated), double-stranded RNA (also different from the vertebrate RNA, which is single-stranded), peptidoglycan and lipoteichoic acid, also had similar properties to LPS (for review see^111^). The structure of lipid A was described in 1983, and the process for its synthetic production, which allowed for the generation of a pure solution of the substance, was discovered in 1985.^112^ ^,^ ^113^

In 1989, there was a paradigm shift in immunology. Janeway introduced the idea that the primary factor that stimulates the initiation of an immune response is the presence of molecules in microorganisms capable of activating the innate immune system.^3^ This activation would occur through receptors of the innate immune system responsible for recognizing molecules that have been evolutionarily conserved in a broad range of microorganisms but are not present (*i.e.*, “foreign”) in the host organism (non-self, foreign molecules). Janeway named these receptors molecular pattern recognition receptors and suggested that molecules such as LPS are one of their potential ligands.^3^ ^,^ ^114^ It is impressive that, at the time, only one paper showing the presence of a mannose binding lectin, synthesized in the human liver and released into the blood, was available to confirm this hypothesis and the existence of pattern recognition receptors.^115^

Seventeen years later, Ephraim Fuchs and Poli Matzinger revised the concept that non-self-foreign molecules are uniquely responsible for activating receptors of the innate immune system.^116^ Fuchs and Matzinger argued that during an infection, there is tissue destruction and the release of molecules that are naturally not exposed to the extracellular environment and that these molecules are responsible for providing a “danger signal” by activating the innate immune system in a manner similar to non-self-foreign molecules. They thus created the concept of danger-associated molecular patterns, changing the focus from Janeway’s activation of the innate immune system by “non-self-foreign” to “danger” signals. This change explains why our bodies tolerate commensal bacteria or why a mother’s body does not attack and destroy a developing fetus during pregnancy. However, the danger theory still has strong opponents in the scientific community. These opponents argue that the concept of “danger” is too vague, given that cells die because of physical destruction all the time in our bodies without causing immune responses.

The search for the LPS receptor and other pattern recognition receptors progressed slowly until 1996, when the group led by Jules Hoffman (1941-) showed that flies of the genus *Drosophila* needed specific genes called Toll genes to combat fungal infections.^117^ This seminal work was the first to suggest that Toll genes were involved in immune responses. Bruno Lemaitre et al. showed that, after microbial infection, expression of the antifungal peptide *Drosomycin* was upregulated following activation of the Toll pathway. A year later, Ruslan Medzhitov, a post-doctoral fellow at Janeway’s laboratory, cloned and characterized a human homolog of the *Drosophila Toll* gene.^118^ The expression of this gene was initially named *hToll* by the authors at the time. They demonstrated that transfection of human monocytes with a constitutively active CD4-*hToll* chimeric protein led to activation of nuclear factor kappa B (NF-κB) and expression of the gene encoding CD80 (also known as B7-1). As CD80 was known to be able to provide co-stimulation to T cells, this highly important finding also provided a link between innate and adaptive immunity, fulfilling the criterion that had been postulated by Janeway for the identification of pattern recognition receptors.^111^ Medzhitov and Janeway’s study was followed the next year by the demonstration of five mammalian Toll homologues that were named Toll-like receptors (TLRs) - these included *hToll* which was renamed as TLR4.^119^

On the same year (1998), Ruey-Bing Yang and colleagues^120^ demonstrated that cell cultures become responsive to LPS when toll-like receptor 2 (TLR2) is expressed on the cell membrane. The work by Ruey-Bing Yang is important because it demonstrated for the first time that a toll-like receptor could respond to a toxin derived from microorganisms, *i.e.*, it indicated that TLR2 was a pattern recognition receptor. However, their study was partially flawed, as it was subsequently shown that these cells only responded to the endotoxin preparation used because it was contaminated with lipoprotein, a substance described as a TLR2 ligand. Later that same year, the group lead by Bruce Beutler^121^ showed that the gene responsible for resistance to LPS in C3H/HeJ animals encoded the TLR4 receptor, a finding that was confirmed the following year by other groups.^122^ ^,^ ^123^ Jules Hoffman and Bruce Beutler received the Nobel Prize in Physiology or Medicine in 2011 for their discoveries relating to the activation of innate immunity. Subsequent studies demonstrated that TLR4 is not able to bind to LPS by itself but requires the help of another molecule called MD2, which also binds to LPS, forming a LPS-MD2 complex that is then able to bind to the receptor.^124^ The definitive characterization showing that the LPS-MD2 complex binds to TLR4 was achieved in 2009 using crystallization techniques.^125^

Currently, 10 Toll-like receptors have been described in humans and 12 have been described in mice; their signaling pathways have already been characterized, and various molecules derived from microorganisms have been shown to be ligands for these receptors. In addition, other families of molecular pattern recognition receptors have also been discovered, such as NOD-like receptors (NLRs), RIG-I-like receptors (RLRs) and C-type lectin receptors (CLRs), among others. It has also been shown that host molecules are capable of binding to pattern recognition receptors. For example, host nucleic acids (DNA and RNA) can bind to TLR7, TLR8 and TLR9 (for review see^126^), and heme, a component of hemoglobin, is able to bind to TLR4.^127^

This brief historical overview, which leaves out several authors, events and studies, (a) highlights the importance and brilliance of the protagonists; (b) allows us to understand how, in just over a century, the concept of innate immunity has developed and changed from a sub-area of toxicology to an ever-expanding branch of biological science and (c) helps us to remember that a theory that can explain all known phenomena of innate immunity does not currently exist and that perhaps a paradigm shift is needed.
